# Complete plastome sequence of *Paeonia lactiflora* Pall. (Paeoniaceae: Saxifragales)

**DOI:** 10.1080/23802359.2018.1501311

**Published:** 2018-10-17

**Authors:** Tahir H. Samigullin, Maria D. Logacheva, Galina V. Degtjareva, Sergey V. Efimov, Elena I. Terentieva, Carmen M. Vallejo-Roman

**Affiliations:** a A. N. Belozersky Institute of Physico-Chemical Biology, Lomonosov Moscow State University, Moscow, Russia;; b Biological Faculty, Lomonosov Moscow State University, Moscow, Russia

**Keywords:** Illumina sequencing, *Paeonia lactiflora*, phylogenetic analysis, plastome

## Abstract

*Paeonia lactiflora* has been listed as an Endangered species in Russian Federation. The complete plastome was assembled from Next-Generation Sequencing data. It is 152,747 bp in length. It consists of a pair of Inverted Repeat regions (25,651 bp), separated by a small single copy region of 17,033 bp and a large single copy region of 84,412 bp. The plastome encoded 128 genes, including 83 protein coding genes, 37 tRNA, eight rRNA genes, four pseudogenes, and is characterized by loss of the *rpl32* and *infA* genes. Phylogenetic analysis of Paeoniaceae plastomes revealed that *P. lactiflora* clustered with Eurasian peonies (section Paeoniae).

*Paeonia lactiflora* (Pallas 1776) is an important ornamental plant; it also has a long history of use as traditional medicinal and oil plant (Tsai et al. 2008; Zhou et al. 2011). This species grows in meadow slopes and foothills in China, Korea, Mongolia, and the Russian Federation (Far East, Siberia). *P. lactiflora* has been assessed as Endangered to the Russian Far East (Red data book Primorsky Krai 2008; The Red Book of the Russian Federation (plants and fungi) 2008). Climate fluctuation and the reduction of occupied area has resulted in the increasingly declining wild populations of the *P. lactiflora* mature individuals. The comprehensive genomic information is very important to formulate effective conservation strategies for this valuable wild peony species. Here, the complete plastome of *P. lactiflora* was determined using next-generation sequencing technology. The assembled and annotated plastome was deposited in GenBank under the accession number MG897127.

*Paeonia lactiflora* plants were collected in Primorsky Krai, Russia (43 16.512' N, 134 03.144' E.). A voucher specimen (41-3 MW) was deposited in the herbarium of MSU, Russian Federation.

Total DNA was extracted using the cetyltrimethylammonium bromide (CTAB) method (Doyle and Doyle 1987). The total DNA (1 μg) was used to build the paired-end libraries. The library was sequenced using an Illumina HiSeq 2000 instrument (Illumina, Inc., USA). The resulting readings were processed using the CLC Genomics Workbench software v. 5.5. (http://www.clcbio.com). The assembled plastome sequence was annotated using the online tool CpGAVAS (Liu et al. 2012) with default options and then checked manually.

The plastome of *P. lactiflora* is 152,747 bp in length consisting of a pair of inverted repeat regions (25,651 bp) separated by a small single copy region (17,033 bp) and a large single copy region (84,412 bp). The plastome encoded 128 genes, including 83 protein-coding genes, 37 tRNA, eight rRNA genes, and four pseudogenes: *rps19, ycf1, rps18, rpl22* and is characterized by loss of the *rpl32* and *infA* genes. The overall GC content is 38.4%.

The phylogenetic tree was reconstructed using 10 published complete Paeoniaceae and two Saxifragales plastomes as the outgroup. The IRa region was removed from analysis, sequences were aligned using MUSCLE (Edgar 2004), and variable and gap-rich positions were removed from alignment using GBLOCKS (Castresana 2000). Phylogeny inference was performed applying the Bayesian approach using MrBayes (Ronquist et al. 2012). Phylogenetic analysis produced a fully resolved tree with *P. lactiflora* clustered with Eurasian peonies (section Paeoniae) (Figure 1).

**Figure 1.**
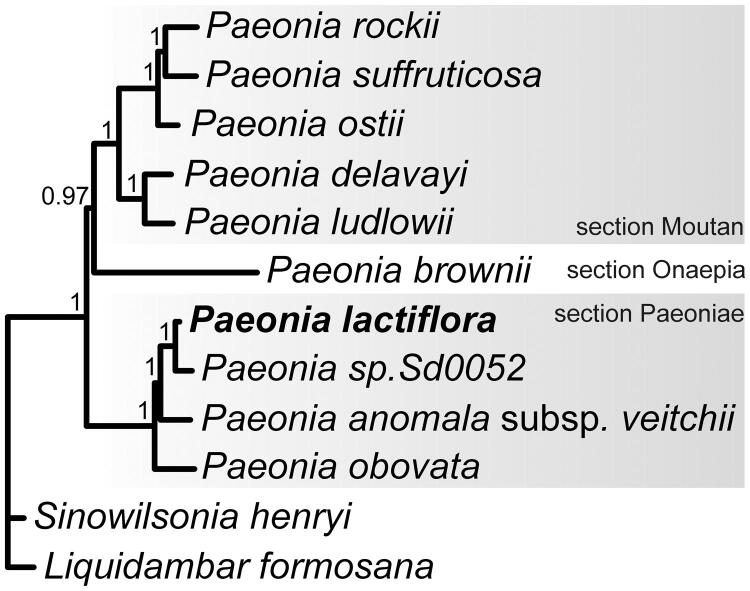
Bayesian tree based on 12 complete plastome sequences. Accession numbers: *P. lactiflora* (MG897127, this study), *Paeonia* sp. Sd0052 (KF753636), *P. anomala* subsp. *veitchii* (NC_032401), *P. obovata* (NC_026076), *P. ludlowii* (NC_035623), *P. delavayi* (NC_035718), *P. ostii* (NC_036834), *P. suffruticosa* (MH_191384*), P. rockii* (MF488719), *P. brownii* (MH191385), *Sinowilsonia henryi* (NC_036069), and *Liquidambar formosana* (NC_023092).

## References

[CIT0001] Castresana J 2000 Selection of conserved blocks from multiple alignments for their use in phylogenetic analysis. Mol Biol Evol. 17:540–552. 10742046 10.1093/oxfordjournals.molbev.a026334

[CIT0002] Doyle JJ, Doyle JL 1987 A rapid DNA isolation procedure for small quantities of fresh leaf tissue. Phytochem Bull. 19:11–15.

[CIT0003] Edgar RC 2004 MUSCLE: multiple sequence alignment with high accuracy and high throughput. Nucleic Acids Res. 32:1792–1797. 15034147 10.1093/nar/gkh340 PMC390337

[CIT0004] Liu C, Shi L, Zhu Y, Chen H, Zhang J, Lin X, Guan X 2012 CpGAVAS, an integrated web server for the annotation, visualization, analysis, and GenBank submission of completely sequenced chloroplast genome sequences. BMC Genomics. 13:715. 23256920 10.1186/1471-2164-13-715 PMC3543216

[CIT0005] Pallas PS 1776 Reise durch Verschiedene Provinzen des Russischen Reichs. St. Petersburg: Kaiserliche Academie der Wissenschaften

[CIT0006] Red Data Book Primorsky Krai 2008 Plants rare and Endangered species of plants and fungi. Official edition Vladivostok p. 688 (in Russian).

[CIT0007] Ronquist F, Teslenko M, van der Mark P, Ayres DL, Darling A, Höhna S, Larget B, Liu L, Suchard MA, Huelsenbeck JP 2012 MrBayes 3.2: efficient Bayesian phylogenetic inference and model choice across a large model space. Syst Biol. 61:539–542. 22357727 10.1093/sysbio/sys029 PMC3329765

[CIT0008] The Red Book of the Russian Federation (plants and fungi) 2008 The Ministry of Natural Resources and Environment of Russian Federation. Moscow: KMK Scientific Press Ltd p. 885 (in Russian).

[CIT0009] Tsai H-Y, Lin H-Y, Fong Y-C, Wu J-B, Chen Y-F, Tsuzuki M, Tang Ch H 2008 Paeonol inhibits RANKL-induced osteoclastogenesis by inhibiting ERK, p38 and NF-kappaB pathway. Eur J Pharmacol. 588:124–133. 18495114 10.1016/j.ejphar.2008.04.024

[CIT0010] Zhou C, Zhang Y, Sheng Y, Zhao D, Lv S, Hu Tao J 2011 Herbaceous peony (*Paeonia lactiflora* Pall.) as an alternative source of oleanolic and ursolic acids. Int J Mol Sci. 12:655–667. 21340006 10.3390/ijms12010655 PMC3039972

